# ADRENAL CARCINOMA IN CHILDREN: LONGITUDINAL STUDY IN MINAS GERAIS, BRAZIL

**DOI:** 10.1590/1984-0462/;2019;37;1;00002

**Published:** 2018-07-26

**Authors:** Nonato Mendonça Lott Monteiro, Karla Emília de Sá Rodrigues, Paula Vieira Teixeira Vidigal, Benigna Maria de Oliveira

**Affiliations:** a Universidade Federal de Minas Gerais, Belo Horizonte, MG, Brasil.

**Keywords:** Adrenocortical carcinoma, Children, Clinical analysis, Carcinoma adrenocortical, Crianças, Análise clínica

## Abstract

**Objective:**

To analyze clinical, laboratory and histopathological features and the path to diagnosis establishment and treatment of patients with adrenal carcinoma (AC).

**Methods:**

Retrospective study with 13 patients assisted at the pediatric oncology service of Hospital das Clínicas, Universidade Federal de Minas Gerais, Brazil, between 2004 and 2015.

**Results:**

Age at diagnosis ranged from 1.0 to 14.8 years (median: 2.0 years). Manifestations of hypercortisolism were identified in all cases and virilization in all girls. All patients met the Weiss criteria to AC histopathological diagnosis. Immunohistochemistry was performed in 61.5% of the cases. Most patients had stage I disease (76.9%). All subjects were submitted to total tumor resection. Two patients (stages III and IV disease) received chemotherapy associated to mitotane. The only death case was that of a patient with stage IV disease. The probability of overall survival for the entire group up to 5.0 years was 92.3±7.4%. The median time between the onset of symptoms and diagnosis was 9.5 months, and 6.0 months between first visit and start of treatment.

**Conclusions:**

Low age at diagnosis, predominance of cases with localized disease and complete tumor resection - with only one case of tumor capsule rupture - can possibly explain the favorable evolution of the studied population. The long period between onset of symptoms and diagnosis highlights the importance of training pediatricians for early recognition of AC signs and symptoms.

## RESUMO

**Objetivo:**

Analisar as características clínicas, laboratoriais e histopatológicas e o percurso até o estabelecimento do diagnóstico e do tratamento de pacientes com carcinoma de suprarrenal (CSR).

**Métodos:**

Estudo retrospectivo com 13 pacientes tratados no serviço de oncologia pediátrica do Hospital das Clínicas da Universidade Federal de Minas Gerais (HC-UFMG) entre 2004 e 2015.

**Resultados:**

A idade ao diagnóstico variou de 1,0 a 14,8 anos (mediana: 2,0 anos). As manifestações de hipercortisolismo foram identificadas em todos os casos, e as de virilização, em todas as meninas. Todos os pacientes preencheram os critérios de Weiss para diagnóstico histopatológico de CSR. A imuno-histoquímica foi realizada em 61,5% dos casos. A maioria dos pacientes apresentou doença em estádio I (76,9%). Todos foram submetidos à ressecção tumoral total. Dois pacientes (estádios III e IV) receberam quimioterapia associada ao mitotano. O único óbito observado foi do paciente com doença em estádio IV. A probabilidade de sobrevida global para todo o grupo aos 5,0 anos foi de 92,3±7,4%. A mediana de tempo entre o início dos sintomas e o diagnóstico foi de 9,5 meses, e de 6,0 meses entre a primeira consulta e o início do tratamento.

**Conclusões:**

A baixa idade ao diagnóstico, o predomínio de casos com doença localizada e a ressecção tumoral completa - com apenas um caso de ruptura de cápsula tumoral - são possivelmente a explicação para a evolução favorável da população estudada. O longo percurso entre o início dos sintomas e o diagnóstico sugere a importância da capacitação dos pediatras para o reconhecimento precoce dos sinais e dos sintomas do CSR.

## INTRODUCTION

Adrenal carcinoma (AC) accounts for 0.2% of childhood and adolescent malignancies, with annual worldwide incidence of 0.2 to 0.3 cases per million subjects.^1^ ^,^ ^2^ It is more common in females, with 2:1 proportion.^3^ ^,^ ^4^ ^,^ ^5^ Most symptomatic patients present virilization due to increased secretion of androgens or Cushing’s syndrome (hypercortisolism).^6^

In Brazil, regions South and Southeast have 10 to 15 times higher incidence of AC compared to worldwide, reaching 4.2 cases per million inhabitants. This finding seems to be associated with the high prevalence of germline TP53 R337H mutation in the population, being identified in more than 90% of CA cases in these regions.^7^ ^,^ ^8^

The rarity of this disease, the differential diagnosis with other diseases commonly found in pediatric age groups, and frequent diagnosis delay justify the conduction of studies that contribute to the understanding of this neoplasm and increase its dissemination among pediatricians and specialists.^3^

The aim of this study was to analyze clinical, laboratory and histopathological characteristics of patients diagnosed with AC who were being assisted at the pediatric oncology service of a public university hospital, and to understand the path to diagnosis establishment and treatment initiation.

## METHOD

Retrospective longitudinal study whose inclusion criteria were patients aged up to 17 years and 11 months, with diagnosis of AC, and admitted to the pediatric oncology service of Hospital das Clínicas of the Federal University of Minas Gerais (HC-UFMG) from June 2004 to June 2015.

This project was approved by the Research Ethics Committee of UFMG on September 11, 2014, CAAE 32898514.2.00000.5149. Legal representatives of patients recruited were required to sign the informed consent form (ICF).

Demographic, clinical, laboratory, imaging data and information about patient’s situation upon data analysis (alive in follow-up, obit, and loss to follow-up) were obtained by reviewing the patients’ medical records.

To identify the primary tumor site and evaluate the extent of the disease, computed tomography (CT) of the thorax and abdomen, abdominal ultrasonography and/or magnetic resonance imaging (MRI) were used. Bone metastases were investigated by bone scintigraphy. To check for presence and extent of vena cava tumor thrombus, ultrasonography and Doppler echocardiography were performed.

The anatomicopathological reports and the slides used for diagnosis were reviewed to confirm nine Weiss criteria, involving microscopic features: nuclear grade III, nuclear grade IV, mitotic index >5/50 high power fields (HPF), presence of atypical mitoses, clear cells composing up to 25% of the tumor, diffuse architecture, venous invasion, sinusoidal invasion, and tumor capsule invasion.^9^ ^,^ ^10^ ^,^ ^11^ ^,^ ^12^ Tumor weight (in grams) and macroscopic characteristics (extracapsular involvement and macroscopic necrosis) were also evaluated according to criteria suggested by Sandrini, Ribeiro and DeLacerda, later modified by Pereira et al.^7^ ^,^ ^13^

During the study, histopathological review and selection of viable paraffin blocks for immunohistochemical study using Ki-67 and P53 antibodies were performed. Immunohistochemical reactions followed the streptavidin-biotin-peroxidase technique.^14^ Positive and negative controls were used to attest to the fidelity of reactions for all markers. Immunolocalization analysis was conducted by a pathologist, using a Nikon light microscope Eclipse E 200 (400x).

Staging establishment was based on the studies by Pereira et al.^7^, namely:

1. completely resected tumor weighing <200g and absence of metastases;
2. completely resected tumor weighing >200g;
3. macroscopic or unresectable residual tumor or presenting capsule rupture during surgery;
4. distant metastasis.

Information about surgical procedures and other registered treatments (such as chemotherapy with cisplatin, doxorubicin and etoposide associated with mitotane) were also collected.

Registration number and specialty of consulted doctors seen until diagnosis were collected, as well as the time between onset of symptoms and first consultation, waiting time for an appointment with a specialist, diagnosis and treatment initiation (date of surgical resection).

Descriptive statistical analysis was made to obtain absolute and relative frequencies, medians, means and standard deviations (SD). To estimate overall survival, the Kaplan Meier method was applied. The “event” considered for the analysis was obit. Patients who did not present an event were censored at the date of analysis of results. The one patient who abandoned the treatment - loss to follow-up - was censored on the date of last recorded visit.

## RESULTS

The sample held 13 patients consecutively admitted to the service in the study period. Demographic characteristics are described in Table 1.

**Table 1:** Demographic characteristics of the study population: 13 patients diagnosed with adrenal carcinoma.

Characteristic	Median	Min-Max
	n	%
Age upon diagnosis (years)	2.0	1.1-14.8
Time of follow-up (years)	4.6	0.4−10.5
Gender		
Female	10.0	76.9
Male	3.0	23.1
Origin		
Belo Horizonte	4.0	30.8
Minas Gerais countryside	9.0	69.2
Family history of neoplasms		
Yes	5.0	38.5
No	8.0	61.5
Outcome		
Alive and free of disease	11.0	84.6
Obit	1.0	7.7
Lost to follow-up	1.0	7.7

The clinical manifestations presented at diagnosis are listed in Table 2. Hirsutism and virilization were identified as two of the initial signs that led patients to the first medical appointment in 54.0% and 38.5% of cases, respectively.

**Table 2:** Clinical manifestations upon diagnosis of 13 patients with adrenal carcinoma.

Sign/symptom	n	%
Terminal hairs appearance	13	100.0
Pubertal progress	13	100.0
Hypercortisolism clinical manifestations	13	100.0
Virilization*	10	100.0
Enlarged clitoris*	10	100.0
Acne	9	69.2
Systemic arterial hypertension	5	38.5
Cushingoid facies	4	30.8
Weight gain	3	23.1
Voice changes	3	23.1
Abdominal pain	2	15.4
Fever	1	7.7
Muscle hypertrophy	1	7.7

*Females (n=10).

Tumors were identified by CT scan and/or abdominal ultrasound. In most patients (61.5%), both examinations were performed. MRI was noted in only one patient’s record. Left gland involvement was slightly prevalent, accounted for in 53.8% of cases. No patient presented bilateral tumor.

Most patients (69.2%) were submitted to Doppler echocardiography; all of them had normal results. Only three patients went through bone scintigraphy, which did not show alterations. Thoracic CT scans performed (61.5%) did not show abnormalities. Of the eight patients with bone age evaluation upon diagnosis written down in their medical record, seven had indication of bone age advancement.

Table 3 addresses preoperative hormonal evaluation, noting that some tests were performed in different laboratories before the patients were admitted to the institution where the study was conducted, which resulted in different reference values for different methods. Therefore, the results were not specified and we chose to use the terms “increase” and “increased” when it came to reference values.

**Table 3:** Preoperative hormonal characteristics of the 13 patients with adrenal carcinoma.

Patient	Gender	Age upon diagnosis (year/month)	Serum cortisol	Urinary cortisol	DHEA-S	Testosterone	Androstenedione	17-OH-progesterone	Aldosterone	Serum renin
1	F	2y3m	N	0	I	I	I	I	0	0
2	F	1y	N	0	I	I	0	0	0	0
3	F	1y10m	N	I	I	I	0	I	0	0
4	M	1y	N	0	I	N	I	I	0	0
5	F	2y	N	0	I	I	0	I	I	0
6	F	3y	0	0	I	I	I	I	0	0
7	M	14y9m	I	I	0	0	0	0	0	0
8	F	1y6m	N	0	I	I	I	I	0	0
9	F	7y6m	I	0	I	I	0	N	0	0
10	F	13y1m	I	0	N	I	I	I	I	0
11	F	1y5m	N	0	I	N	I	I	0	0
12	M	4y10m	N	0	I	I	I	0	0	0
13	F	1y6m	N	0	N	0	N	0	0	0

F: female; M: male; N: normal compared to reference value; 0: not informed; I: increased compared to reference value; DHEA-S: Dehydroepiandrosterone sulfate.

All patients met the Weiss criteria for anatomopathological diagnosis of AC (median: six criteria). They all had atypical mitoses and 8 (61.5%) had mitotic index >5/50 high power fields.

Tumor weight ranged from 5 to 1040 g (median: 25 g). Three patients with the highest tumor weights were classified in stages II (300 g), III (1,040 g) and IV (300 g). Immunohistochemical study was possible for eight patients, with Ki-67 marker indicating positivity from 0.0 to 60.0% (median: 15.0%). The P53 marker was positive in 87.5% of cases analyzed.

Eleven patients had localized disease (stages I and II), most of them aged up to 4 years (nine cases). One patient was classified as stage III and one as stage IV, both aged above 4 years. Metastasis was identified only in patient 7 (liver, stage IV). Eight of the 13 patients (61.5%) had a record of absence of vena cava thrombus and, in 38.5% of cases, this parameter was not reported.

All patients went through complete tumor resection by laparotomy. Tumor capsule rupture happened in only one case (patient 9), whose tumor had the highest weight among all (1,040 g). Patients with advanced-stage disease (patients 7 and 9) received mitotane-associated chemotherapy. For the others, surgical resection was the only therapeutic modality employed. The only death occurrence was that of a patient in stage IV (patient 7), due to neoplastic progression (Table 4). Estimated overall survival probability for the whole group was 92.3±7.4% after 5 years of follow-up.

**Table 4:** Characterization of the 13 patients diagnosed with adrenal carcinoma as to clinical variables and outcome.

Age	Patient #	Age upon diagnosis	Staging	Time of follow-up (year/month)	Outcome
≤4 years	2	1y	II	9y9m	AND
	4	1y	I	6m	LF
	11	1y5m	I	4y7m	AND
	8	1y6m	I	2y	AND
	13	1y6m	I	8m	AND
	3	1y10m	I	9y9m	AND
	5	2y	I	7y11m	AND
	1	2y3m	I	10y6m	AND
	6	3y	I	7y9m	AND
>4 years	12	4y10m	I	9m	AND
	9	7y6m	III	4y9m	AND
	10	13y1m	I	4y9m	AND
	7	14y9m	IV	4m	Obit

LF: loss to follow-up; AND: alive and free of disease.

The number of physicians who evaluated patients before diagnosis ranged from three to five (median: 4), considering different specialties. Twelve patients had their first appointment with a pediatrician, and one patient with an endocrinologist. The median time between onset of symptoms and first medical appointment was one month. Regarding the interval between the beginning of symptomatology and specialized consultation, whether with a pediatric endocrinologist or pediatric oncologist, the median was six months, 25 months at the latest. As for the time between onset of symptoms and diagnosis, the median was 9.5 months, and between first medical visit and confirmation of diagnosis, 7.5 months.

Considering that tumor resection is a therapeutic modality, the median time interval between first consultation and beginning of treatment was six months. All tumor resections were performed by a pediatric surgeon experienced in oncology. With the patient’s admission to the HC-UFMG pediatric oncology service, the maximum time elapsed to perform tumor resection was 4.0 weeks (median: 1.5 weeks), and, for diagnosis confirmation through anatomicopathological examination’s result, 9.0 weeks (median: 3.5 weeks).

## DISCUSSION

The present study dealt with an 11-year experience at a single reference center for the treatment of children with a diagnosis of AC, being the first report in the state of Minas Gerais. The clinical and laboratory characteristics of this series were similar to those of other studies performed in national and international centers. All patients presented pubertal progression at diagnosis, and all girls evolved with virilization. Although cancer is a rare condition in childhood, it is an important cause of early puberty, and AC should always be considered in differential diagnosis.^15^ ^,^ ^16^

In spite of the importance of hormonal dosages for diagnosis, evaluation of tumor functionality and early detection of recurrence, in this study we analyzed data regarding evaluations in the preoperative period.^7^ ^,^ ^17^ Although serum cortisol was normal in most cases, all patients had clinical signs of Cushing’s syndrome. One hypothesis for this finding is the possibility of “false negative” results, since isolated doses of serum cortisol are not very useful to diagnose hypercortisolism, the ideal tests being free cortisol measurement in 24-hour urine or low dexamethasone suppression test.^7^ ^,^ ^18^ Only two patients had urinary cortisol dosage, and both presented values ​​above reference for this method. Increased levels of Dehydroepiandrosterone sulfate (DHEA-S) are laboratory findings suggestive of AC presence, but alterations in testosterone, 17-OH progesterone and androstenedione levels are also reported.^5^ ^,^ ^7^ We found elevation of these hormones in most patients studied.

Abdominal CT scan or MRI are recommended for the diagnosis of primary tumor and the evaluation of abdominal metastases.7,13,19 Chest CT scan is the exam of choice to investigate lung metastases, since MRI has low sensitivity in detecting these metastases. Presence of venous thrombus is described in the literature as a poor prognostic factor, and the tests indicated for its investigation are Doppler echocardiogram and abdominal ultrasound with Doppler.7,13,19,20 As recommended, most patients were submitted to abdominal CT scan as part of the initial evaluation, but no chest CT scan, Doppler echocardiogram, or Doppler abdominal ultrasound data were found in a significant number of patients’ records - noting that no record does not represent certainty of non-performance of such exams.

Because of the rarity of AC and its heterogeneous behavior, it is a challenge to establish prognostic factors for this disease.^4^ ^,^ ^21^ ^,^ ^22^

The importance of histology for prognosis is controversial.^19^ Although adrenocortical tumors can be adequately classified as per Weiss or Van Slooten scores^23^ in the adult population, there is still no consensus on the criteria to define histological malignancy characteristics when these tumors affect patients in pediatric age.^5^ ^,^ ^23^ All patients met the Weiss criteria for anatomopathological diagnosis of AC. The Weiss system^9^ provides specific guidelines for distinguish adenoma from adrenal carcinoma and is considered the standard to state malignancy in adrenal cortex tumors, although it is not reliable when it comes to children. The characteristics considered most important by most authors are mitotic index and presence of atypical mitoses, being not only important criteria for diagnosis of malignancy, but also for survival prediction.^24^ ^,^ ^25^ Tumor weight is considered another determining factor of malignancy.^13^ ^,^ ^26^ ^,^ ^27^ In this study, patients with higher tumor weight were in more advanced stages compared to the rest of the group.

Although immunohistochemistry may be useful in differentiation between adrenal neoplasms and other tumors, there is still no consensus as to its importance as a prognostic indicator in AC.^28^ The immunohistochemical staining for P53 is considered a useful indicator for TP53 gene mutation analyses in several types of neoplasms; however, it is not possible to establish a perfect correlation between the presence of mutation and immunohistochemical expression. There are no studies that validate this method as a substitute or alternative for mutation identification in AC cases. Several papers have shown the diagnostic and poor-prognosis value of Ki-67 expression >10%.^5^ ^,^ ^19^ In this series, patients with stage III and IV disease had 50 and 60% positivity Ki-67, respectively.

Recent studies indicate the need for a review of the staging system initially proposed by Pereira et al.,^7^ which was used in this study, in order to include and give emphasis to factors related to unfavorable prognosis such as age greater than or equal to four years, extension of primary disease to adjacent structures, and presence of metastases. For these authors, the development and validation of more robust staging systems could help identify patients who can benefit from more intensive treatments.^22^ In our sample, patients with advanced-stage disease were older than four years, and those presenting with metastasis died. The findings related to the latter are also in agreement with McAteer et al.’s report, as they state that older children present with disease similar to that of adults, with a survival rate of 30 to 40% in five years.^4^

According to the literature, complete tumor resection without capsule rupture is the therapy with the highest cure rate, which is in consonance with the results of this study.^21^ ^,^ ^29^ All patients in our sample were submitted to laparotomy. Video-surgery was not recommended to prevent tumor rupture.

Despite the lack of consensus on the efficacy of the adrenolytic agent mitotane and cytotoxic chemotherapy in pediatric patients, these therapeutic modalities have been indicated in cases where tumor resection is not possible or there is metastatic disease.^5^ ^,^ ^29^ Retrospective studies conducted with adult patients suggest that adjunct treatment with mitotane may benefit patients with a Ki-67 index greater than 10%.^5^ In this study, only patients with advanced disease (stages III and IV) received chemotherapy combined with mitotane, and both had a Ki-67 index surpassing 10%.

The relatively small number of patients in the sample did not allow survival rate analyses according to age, disease staging, tumor size and other features indicated in the literature as prognostic factors in AC;^5^ ^,^ ^22^ however, the estimated overall survival probability was higher than reported in several national and international studies.^5^ ^,^ ^22^ This favorable progress in survival is possibly explained by the predominance of patients aged less than 4 years upon diagnosis, by the predominance of cases with localized disease, and by all patients being submitted to complete tumor resection, factors associated with better prognosis in the literature.^5^

The median time between first medical visit and diagnosis (7.5 months), and between onset of symptoms and diagnosis confirmation (9.5 months) was higher than that reported in a study conducted with 125 children in the State of Paraná, with median time of 6.0 months and mean time of 11.1 months.^7^ However, in a review including 21 papers from different countries, Liou and Kay identified a systematic delay in diagnosis, with time between onset of symptoms and tumor diagnosis ranging from 3 days to 66 months (mean 10.6 months).^30^ The delay in diagnosis^31^ is considered the main reason for disease staging advance and low survival rates reported in the literature.^1^ ^,^ ^3^ ^,^ ^4^ ^,^ ^5^

In this study, the number of physicians who evaluated patients before diagnosis ranged from three to five. Most of the patients had their first consultation with the pediatrician, which reinforces the importance of this professional’s qualification for early identification of warning signs and consequent diagnosis of neoplasms in the pediatric population, once these are often similar to clinical manifestations common to other diseases more common in this age group.^2^ ^,^ ^5^ ^,^ ^19^

The possibility of radical tumor resection - first choice treatment for AC - is related to the time interval between the onset of symptoms and diagnosis.^30^ In this study, the median interval between the first medical appointment and the surgical procedure was six months. However, after patients were admitted to the service, diagnosis confirmation and beginning of the treatment took place according to the deadlines established by the Ministry of Health (Ministerial Order 140, February 27, 2014).^32^

The main limitations of this study are related to its retrospective design. The lack of standardization in data registration on medical records and of a storage protocol for material destined to anatomopathological examination contributed to the unavailability of some data. The relatively small sample should also be mentioned, although most studies with more representative casuistries are multicenter by nature, considering the rarity of this disease.^5^ ^,^ ^22^

In the institution where this study was carried out, as well as in most pediatric oncology reference centers across the country, TP53 mutation research is not a routine procedure, and it could aid comparisons with studies involving populations from other States of Brazil, especially Paraná and São Paulo, and also with international reports.^31^

Conclusion is that lower age upon diagnosis in the studied population, as well as the prevalence of localized disease and high rate of complete tumor resection with low capsule rupture rate, may justify the high survival index observed in this study.

The analyses of the path of patients, from the beginning of symptomatology to diagnosis and treatment, suggest difficulty in the recognition of the disease and highlights the importance of qualification for health professionals as to warning signs, especially pediatricians, increasing the index of disease suspicion and contributing to AC early diagnosis.

## References

[1] Figueiredo B, Ribeiro RC (2004). Childhood adrenocortical tumours. Eur J Cancer.

[2] Rodriguez-Galindo C, Figueiredo BC, Zambetti GP, Ribeiro RC (2005). Biology, clinical characteristics, and management of adrenocortical tumors in children. Pediatr Blood Cancer.

[3] Ribeiro RC, Pinto EM, Zambetti GP, Rodriguez-Galindo C (2012). The international pediatric adrenocortical tumor registry initiative: contributions to clinical, biological, and treatment advances in pediatric adrenocortical tumors. Mol Cell Endocrinol.

[4] McAteer JP, Huaco JA, Gow KW (2013). Predictors of survival in pediatric adrenocortical carcinoma: a Surveillance, Epidemiology, and End Results (SEER) program study. J Pediatr Surg.

[5] Kerkhofs TM, Ettaieb MH, Verhoeven RH, Kaspers GJ, Tissing WJ, Loeffen J (2014). Adrenocortical carcinoma in children: First population-based clinicopathological study with long-term follow-up. Oncol Rep.

[6] Pereira RM, DeLacerda L, Lacerda HM, Michalkiewicz E, Sandrini F, Sandrini R (2006). Childhood adrenocortical tumors: a review. Hered Cancer Clin Pract.

[7] Pereira RM, Michalkiewicz E, Sandrini F, Figueiredo BC, Pianovski M, França SN (2004). Childhood adrenocortical tumors. Arq Bras Endocrinol Metab.

[8] Ribeiro RC, Pinto EM, Zambetti GP (2010). Familial predisposition to adrenocortical tumors: Clinical and biological features and management strategies. Best Pract Res Clin Endocrinol Metab.

[9] Weiss LM (1984). Comparative histologic study of 43 metastasizing and nonmetastasizing adrenocortical tumors. Am J Surg Pathol.

[10] Weiss LM, Medeiros LJ, Vickery AJ (1989). Pathologic features of prognostic significance in adrenocortical carcinoma. Am J Surg Pathol.

[11] Weiss LM, Bertagna X, Chrousos GP, Kawashima A, Kleihues P, Koch CA, DeLellis RS, Lloyd RV, Heitz PU, Eng C (2004). Adrenal cortical carcinoma. Pathology and genetics of tumors of endocrine organs.

[12] Lau SK, Weiss LM (2009). The Weiss system for evaluating adrenocortical neoplasms: 25 years later. Hum Pathol.

[13] Sandrini R, Ribeiro RC, DeLacerda L (1997). Childhood adrenocortical tumors. J Clin Endocrinol Metab.

[14] Hsu SM, Raine L, Fanger H (1981). A comparative study of the peroxidase-antiperoxidase method and avidin-biotin complex method for studying polypeptide hormones with radioimmunoassay antibodies. Am J Clin Pathol.

[15] Zwiebel WJ, Murray KA (1995). Imaging assessment of pubertal disorders. Semin Ultrasound CT MR.

[16] Wendt S, Shelso J, Wright K, Furman W (2014). Neoplastic causes of abnormal puberty. Pediatr Blood Cancer.

[17] Menéndez-Calderón MJ, Casal F, Prieto J, Cacho L, Tusón C (2006). Adrenocortical carcinoma. A retrospective analysis of five cases. An Med Interna (Madrid).

[18] Stratakis CA (2012). Cushing syndrome in pediatrics. Endocrinol Metab Clin North Am.

[19] Allolio B, Fassnacht M (2006). Adrenocortical Carcinoma: clinical update. J Clin Endocrinol Metab.

[20] Kasperlik-Zaluska AA, Zgliczynski W, Slapa RZ, Cichocki A (2008). Retroperitoneal hemorrhage from adrenocortical carcinoma as a poor prognostic factor. Int J Biomed Sci.

[21] Michalkiewicz E, Sandrini R, Figueiredo B, Miranda EC, Caran E, Oliveira-Filho AG (2004). Clinical and outcome characteristics of children with adrenocortical tumors: a report from the International Pediatric Adrenocortical Tumor Registry. J Clin Oncol.

[22] Gulack BC, Rialon KL, Englun BR, Kim J, Talbot LJ, Odibe OO (2016). Factors associated with survival in pediatric adrenocortical carcinoma: an analysis of the National Cancer Data Base (NCDB). J Pediatr Surg.

[23] Gomes SM, Amaral CM, Albuquerque GC, Silva FF, Ibiapina GR (2013). Carcinomas adrenocorticais: divergências entre critérios prognósticos. Rev Ciênc Saúde Nova Esperança.

[24] Assié G, Antoni G, Tissier F, Caillou B, Abiven G, Gicquel C (2007). Prognostic parameters of metastatic adrenocortical carcinoma. J Clin Endocrinol Metab.

[25] Volante M, Buttigliero C, Greco E, Berruti A, Papotti M (2008). Pathological and molecular features of adrenocortical carcinoma: an update. J Clin Pathol.

[26] Mondal SK, Dasgupta S, Jain P, Mandal PK, Sinha SK (2013). Histopathological study of adrenocortical carcinoma with special reference to the Weiss system and TNM staging and the role of immunohistochemistry to differentiate it from renal cell carcinoma. J Cancer Res Ther.

[27] Jain M, Kapoor S, Mishra A, Gupta S, Agarwal A (2010). Weiss criteria in large adrenocortical tumors: A validation study. Indian J Pathol Microbiol.

[28] Wieneke JA, Thompson LD, Heffess CS (2003). Adrenal cortical neoplasms in the pediatric population: a clinicopathologic and immunophenotypic analysis of 83 patients. Am J Surg Pathol.

[29] Wajchenberg BL, Pereira MA, Mendonça BB, Latronico AC, Carneiro PC, Alves VA (2000). Adrenocortical carcinoma: clinical and laboratory observations. Cancer.

[30] Liou LS, Kay R (2000). Adrenocortical carcinoma in children. Review and recent innovations. Urol Clin North Am.

[31] Custódio G, Komechen H, Figueiredo FR, Fachin ND, Pianovski MA, Figueiredo BC (2012). Molecular epidemiology of adrenocortical tumors in southern Brazil. Mol Cell Endocrinol.

[32] Brazil - Ministério da Saúde. Secretaria de Atenção à Saúde (2014). Portaria nº 140, de 27 de fevereiro de 2014.

